# Salvage Androgen Deprivation Therapy as Potential Treatment for Recurrence after Robot-Assisted Radical Prostatectomy

**DOI:** 10.3390/cancers16071304

**Published:** 2024-03-27

**Authors:** Hiroshi Kano, Yoshifumi Kadono, Renato Naito, Tomoyuki Makino, Hiroaki Iwamoto, Hiroshi Yaegashi, Shohei Kawaguchi, Takahiro Nohara, Kazuyoshi Shigehara, Kouji Izumi, Atsushi Mizokami

**Affiliations:** 1 Department of Integrative Cancer Therapy and Urology, Kanazawa University Graduate School of Medical Science, Kanazawa 920-8640, Japan; kanazawa_iimati@yahoo.co.jp (H.K.); thealfuu@yahoo.co.jp (R.N.); mackeeen511@gmail.com (T.M.); hiroaki017@yahoo.co.jp (H.I.); hyae2002jp@yahoo.co.jp (H.Y.); shohei_k2001@yahoo.co.jp (S.K.); t_nohara704@yahoo.co.jp (T.N.); kshigehara0415@yahoo.co.jp (K.S.); azuizu2003@yahoo.co.jp (K.I.); mizokami@med.kanazawa-u.ac.jp (A.M.); 2 Department of Nephrology and Urology, Japanese Red Cross Fukui Hospital, Fukui 918-8501, Japan

**Keywords:** robot-assisted radical prostatectomy, recurrence, salvage treatment, intermittent androgen deprivation therapy, testosterone

## Abstract

**Simple Summary:**

The efficacy of intermittent androgen deprivation therapy (ADT) for biochemical recurrence (BCR) after robot-assisted radical prostatectomy (RARP) is unknown, and its usefulness in Japanese practice needs to be investigated. A retrospective analysis of 85 patients who underwent RARP at Kanazawa University Hospital between 2009 and 2019 and were selected for intermittent ADT for postoperative recurrence was analyzed. The 5-year castration-resistant prostate-cancer-free survival rates, cancer-specific survival, and overall survival were 92.7%, 98.3%, and 94.7%, respectively. A subgroup analysis of 69 patients who completed initial ADT was conducted to evaluate the rate of BCR following initial ADT. The 5-year BCR-free survival rate was 53.2%. Multivariate analysis identified testosterone ≤ 0.03 ng/mL during ADT as the sole predictor of BCR after ADT. ADT may be applicable for BCR after RARP, and strong testosterone suppression could lead to better outcomes.

**Abstract:**

Background: The efficacy of intermittent androgen deprivation therapy (ADT) for biochemical recurrence (BCR) after robot-assisted radical prostatectomy (RARP) is unknown, and its usefulness in Japanese practice needs to be investigated. Methods: We conducted a retrospective analysis of 85 patients who underwent RARP and were selected for intermittent ADT for postoperative recurrence at Kanazawa University Hospital between 2009 and 2019. Intermittent ADT was administered for 2 years. If prostate-specific antigen levels increased post-treatment, intermittent ADT was reinitiated. The median follow-up period was 47 months. Results: The 73 patients had completed the initial course of ADT, and 12 were under initial ADT. The 5-year castration-resistant prostate-cancer-free survival rates, cancer-specific survival, and overall survival were 92.7%, 98.3%, and 94.7%, respectively. A subgroup analysis of 69 patients who completed intermittent ADT was conducted to evaluate the BCR rate following initial ADT. The 5-year BCR-free survival rate was 53.2%. Multivariate analysis identified testosterone ≤ 0.03 ng/mL during ADT as the sole predictor of BCR after ADT. Conclusions: Salvage intermittent ADT may be an effective treatment option for BCR after RARP. In addition, it would be useful to confirm strong testosterone suppression as a criterion for transition to intermittent therapy.

## 1. Introduction

Prostate cancer is the most common cancer worldwide [1], and radical prostatectomy (RP) is one of the recommended first-line treatments [2]. However, approximately 15–35% of patients experience postoperative prostate-specific antigen (PSA) elevation and biochemical recurrence (BCR) [3,4,5]. Whether such PSA increases are due to local recurrence or distant metastasis has not been clarified [6]. Salvage radiotherapy (RT) is a standard of treatment but its effectiveness is uncertain because the abnormal PSA levels could be due to local, regional, distant, or combined recurrence [7]. RT is ineffective for distant metastases; thus, androgen deprivation therapy (ADT) is required. ADT combined with RT can prolong cancer-specific survival (CSS) and overall survival (OS), while postoperative ADT has certain advantages [8,9].

The use of salvage ADT for managing recurrence following RP is potentially effective, although its efficacy is supported primarily by retrospective studies [10,11]. An additional problem is that the therapy’s optimal drug protocol and duration have not yet been established. Recently, the efficacy of ADT + Androgen Receptor Signaling Inhibitor use has been shown for BCR, but it is not a general treatment strategy [12]. The estimated 10-year CSS and OS after RP are reported to be 84–93.6% and 67–85.6%, respectively [13,14], and long-term survival is expected. Continuing ADT throughout that survival period is expected to be problematic in terms of overtreatment and medical economics [15]. Therefore, a decision must be made to terminate ADT as a postoperative salvage therapy temporarily. However, the outcome of intermittent ADT for postoperative BCR is not yet known.

Intermittent ADT does not differ significantly in oncological outcomes with continuous ADT and preserves quality of life [16]. In their review, Artibani et al. [11] recommended intermittent ADT when salvage ADT is the treatment of choice. In addition, it was reported that ADT is more likely to be successful in the Japanese population than in other racial groups [17], suggesting that continued ADT may not be necessary. Moreover, long-term ADT is reported to exacerbate cardiac disease and bone fractures [18,19,20], suggesting that it is necessary to limit the duration of ADT. Therefore, intermittent ADT was expected to be a good outcome for BCR after RP.

The present study investigated the usefulness of intermittent salvage ADT for BCR after robot-assisted RP (RARP). In addition, the objective was to identify predictors of BCR in patients after completion of initial ADT and to identify factors that would be criteria for transitioning to intermittent therapy.

## 2. Materials and Methods

Between 2009 and 2019, 453 patients underwent RARP at Kanazawa University Hospital. Among them, 95 patients had recurrence of PSA or distant metastasis. Excluding 10 patients who opted for RT or follow-up for recurrence, 85 patients who were selected for ADT for recurrence were included in this study.

The date of BCR was defined as the date the PSA level first increased to ≥0.2 ng/mL after RARP. In cases where the PSA level never decreased to <0.2 ng/mL after RARP, the date of surgery was defined as the date of BCR. Recurrence included BCR and development of metastases. PSA doubling time was measured retrospectively from the date of PSA recurrence. PSA failure after ADT was defined as a PSA level increase of at least 2.0 ng/mL and a 25% increase from nadir, confirmed using a second PSA test at least 4 weeks later. Cases meeting the above criteria were diagnosed as castration-resistant prostate cancer (CRPC).

ADT included monotherapy with luteinizing hormone-releasing hormone (LH-RH) analogs and combination therapy with anti-androgens and LH-RH analogs. Combination therapy for 2 years was administered, and one course was completed after PSA levels were confirmed to be less than sensitive (<0.008 ng/mL). Failure of intermittent treatment was defined as a rise in PSA level above 0.2 ng/mL after ADT was discontinued. If intermittent therapy failed, ADT was readministered. This treatment strategy was continued until the patients developed CRPC. Adverse events were evaluated using the Common Terminology Criteria for Adverse Events (CTCAE) version 5.0.

Testosterone levels were measured on the last day of LH-RH analog administration for the first course. If testosterone was not measured on that date, the value on the day that was 1 year or more after administration and closest to the last administration date or within 3 months after the last administration was used.

The follow-up for this study was completed in August 2022. The median follow-up period was 47 months. OS, CSS, and CRPC progression-free survival (CFS) were estimated using the Kaplan–Meier method. Survival distributions were compared using the log-rank test. Cox proportional hazards models were used for univariate analysis of CRPC predictors. The observation period was from the beginning of the initial ADT to the time of the event or to the final follow-up.

To analyze the rates of BCR after starting initial ADT, we focused on patients who successfully finished the initial ADT. The duration to the occurrence of BCR after completing ADT was defined as BCR-free survival (BCR-fs) and estimated using the Kaplan-Meier method. Univariate and multivariate analyses were conducted to identify predictors of BCR after initial ADT, and significant differences were analyzed using the Cox proportional hazards model. 

Statistical analyses were performed using commercially available software, including SPSS version 27.0 (SPSS Inc., Chicago, IL, USA) and Prism version 9.4.0 (GraphPad, San Diego, CA, USA). In all analyses, statistical significance was set as *p* value < 0.05. This study was approved by the Institutional Review Board of Kanazawa University Hospital (2020-1223-5).

## 3. Results

Table 1 lists the characteristics of patients. The median age was 69 (65–73) years. The median initial PSA level was 7.8 (5.6–12.1) ng/mL, with 10 patients having a biopsy Gleason grade group (GGG) = 5. Nineteen patients received neoadjuvant ADT. The surgical GGG could not be evaluated in 10 patients due to neoadjuvant ADT. Due to the focus on localized prostate cancer, no cases with preoperative lymph node metastases were identified. Lymph node dissection was not performed in 32 cases. No lymph node metastases were found in 48 cases. However, lymph node metastases were observed in five cases. The postoperative PSA level was undetectable (<0.008 ng/mL) in 37 patients; the median PSA at the start of ADT was 0.269 (0.241–0.330) ng/mL, the median time from RARP to BCR was 19 (9–40) months, and the median PSA doubling time was 7 (4–12) months. Metastatic recurrence was observed in three patients. At the end of the study, 12 patients were undergoing their first course of ADT; 48 patients had no PSA recurrence after the first course of ADT; 21 patients required two or more courses of ADT. In patients requiring more than two courses of ADT, the median duration of nontreatment periods decreased progressively; the first period lasted for a median of 22.8 months, the second for 15.6 months, and the third for 10.6 months. Median PSA was 0.300 ng/mL at the start of the second course, 0.394 ng/mL at the third course, and 0.244 ng/mL at the fourth course. Three cases exhibited CTCAE grade 1 liver dysfunction attributed to bicalutamide, which subsequently improved upon the discontinuation of bicalutamide. None of the patients experienced CTCAE grade 2 or higher adverse events. A total of four patients had CRPC during the first course of ADT, and one patient had CRPC during the third course of ADT. Four deaths occurred during the observation period, two of which were diagnosed as deaths from prostate cancer.

**Figure 1:**
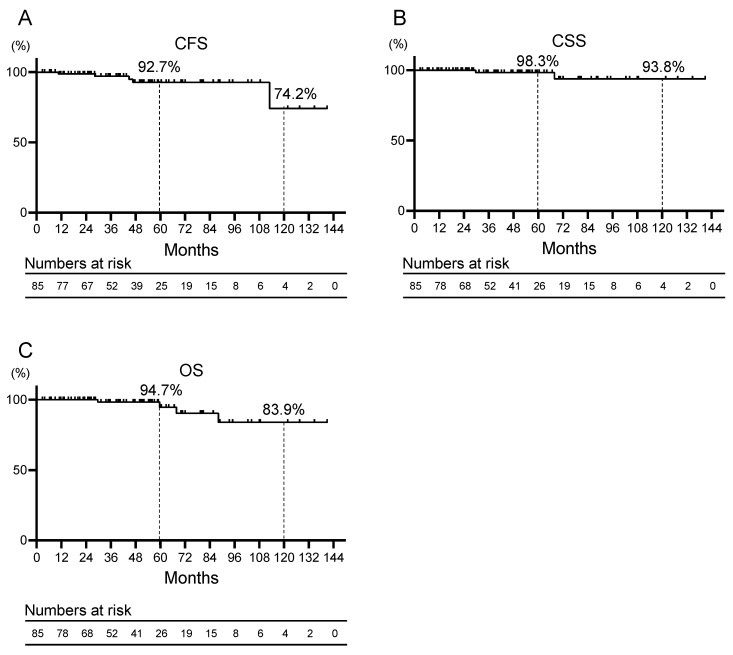
Kaplan–Meier curves for (**A**) castration-resistant prostate cancer progression-free survival (CFS), (**B**) cancer-specific survival (CSS), and (**C**) overall survival (OS) of patients with prostate cancer who received salvage androgen deprivation therapy for recurrence after robot-assisted radical prostatectomy.

The Kaplan–Meier curves for CFS, CSS, and OS are shown in Figure 1A–C. The 5- and 10-year CFS, CSS, and OS rates were 92.7% and 74.2%, respectively; 98.3% and 93.8%, respectively; and 94.7% and 83.9%, respectively. Univariate analysis was performed to identify prognostic factors for CRPC. In the univariate analysis, significant independent predictors of worsening CFS were clinical T stage ≥ 3 (hazard ratio (HR) 10.28, 95% confidence interval (CI) 1.45–73.04, *p* = 0.020), biopsy and surgical GGG = 5 (HR 20.98, 95% CI 2.32–189.76, *p* = 0.007, and HR 27.49, 95% CI 2.91–260.00, *p* = 0.004), PSA at treatment start ≥ 0.5 ng/mL (HR 9.27, 95% CI 1.53–56.05, *p* = 0.015). Multivariate analysis was not performed due to a lack of statistical power (Table 2).

**Figure 2:**
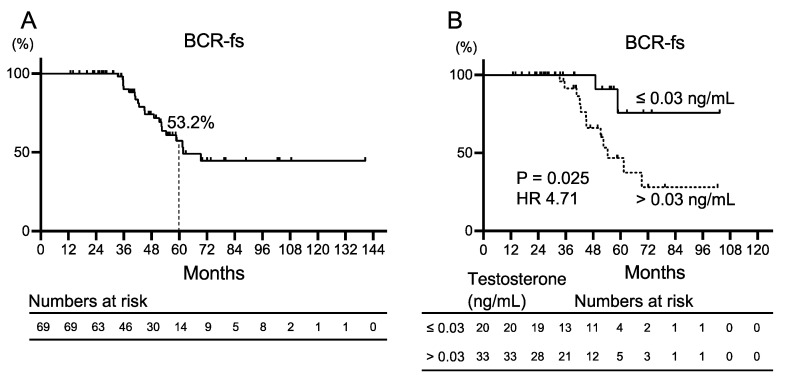
Kaplan–Meier curves for biochemical recurrence-free survival (BCR-fs) (**A**) in patients with prostate cancer who completed initial salvage androgen deprivation therapy for recurrence after robot-assisted radical prostatectomy, and (**B**) patients with testosterone level ≤ 0.03 ng/mL and testosterone level >0.03 ng/mL. HR: hazard ratio.

A subgroup analysis of 69 patients who completed initial intermittent ADT was performed to evaluate the characteristics of patients who were not expected to develop BCR after initial salvage ADT intervention (Table 1). The median age was 68 (64–73) years. The median initial PSA was 8.5 (5.6–12.8) ng/mL, with seven patients with biopsy GGG = 5. The median PSA doubling time was 7 (4–11) months. Testosterone levels were measured in 53 patients, with 20 showing testosterone levels ≤ 0.03 ng/mL near the end of initial ADT and 33 patients > 0.03 ng/mL. Only one patient developed CRPC, and none of the deaths in the subgroup were due to prostate cancer. The Kaplan–Meier curves for BCR-fs are shown in Figure 2A. The 5-year BCR-fs rate was 53.2%. Kaplan–Meier curves for BCR after initial ADT divided by a testosterone level of 0.03 ng/mL are shown in Figure 2B. Patients with testosterone levels >0.03 ng/mL demonstrated significantly worse BCR-fs compared with those with ≤0.03 ng/mL (median BCR-fs, 54 months vs. not reached; HR 4.71, 95% CI 1.64–13.53, *p* = 0.025). Univariate and multivariate analyses were performed to identify factors predicting BCR after initial ADT. In the univariate analysis, a testosterone level ≤ 0.03 ng/mL during ADT was the only factor with a significant difference (HR 4.76, 95% CI 1.06–21.37, *p* = 0.041; Table 3). In patients undergoing RARP, high GGG, and PSA doubling time (PSA-DT) after primary therapy have been reported to be solid prognostic factors for oncologic outcomes [21]. Therefore, in this study, we selected GGG, PSA-DT, and testosterone levels for multivariate analysis. The results revealed that a testosterone level ≤ 0.03 ng/mL during ADT was the only factor that predicted recurrence after ADT treatment (HR 5.10, 95% CI 1.10–23.59, *p* = 0.037; Table 3). 

## 4. Discussion

Adjuvant ADT after RP does not significantly improve OS at the 5-year mark (HR 1.50, 95% CI 0.79–2.85, *p* = 0.35) [22]. Although the efficacy of ADT as an adjuvant postoperatively has been ruled out, its efficacy as salvage remains unresolved. Yokomizo et al. conducted a comparative analysis of outcomes following PSA recurrence after RP in two cohorts: salvage hormone therapy (SHT) with bicalutamide alone and salvage RT (SRT) with or without bicalutamide (SRT ± SHT). The findings revealed a significantly prolonged time to failure (TTF) of bicalutamide in the SRT ± SHT group compared to the SHT group (median TTF 8.6 years vs. 5.6 years; HR 0.56, 95% CI 0.40–0.77, *p* = 0.001). Interestingly, however, no statistically significant disparity was observed between the SHT and SRT ± SHT in clinical relapse-free survival (RFS) (5-year RFS; 93.8% vs. 88.9%; HR 0.90, 95% CI 0.45–1.81, *p* = 0.77) and OS (5-year OS 99.0% vs. 91.4%; HR 1.03, 95% CI 0.46–2.3, *p* = 0.94) [23]. The present study reports similarly excellent CSS (98.3%) and OS (94.7%) at 5 years. This favorable outcome suggests that salvage ADT is one expected option. However, this result may be because the population in this study was Japanese. Fukagai et al. [17] have previously reported that Japanese patients exhibit a response to hormone therapy superior to that of Caucasians. Racial differences have also been noted at the genetic level, with Japanese reported to have a lower frequency of functional single nucleotide polymorphisms (SNPs) that may adversely affect prostate cancer progression during ADT than other races [24,25]. In this context, salvage ADT presents a valuable treatment option for postoperative recurrence in the Japanese population. 

Clinical studies have been performed in the past regarding the outcomes of intermittent and continuous ADT. Hussain et al. performed a comparison of OS between intermittent and continuous ADT treatment for hormone-sensitive prostate cancer with metastases. They found a median OS of 5.8 years in the continuous ADT group and 5.1 years in the intermittent ADT group, but no statistical non-inferiority was demonstrated. On the other hand, intermittent therapy was associated with better erectile function and mental health (*p* < 0.001 and *p* = 0.003, respectively) [16]. A prospective study was conducted to compare intermittent versus continuous ADT for PSA recurrence after RT for localized prostate cancer. Results showed no difference in OS between the two groups. However, intermittent treatment showed potential benefits in physical function, fatigue, dysuria, hot flashes, libido, and erectile function [26]. A retrospective analysis reported that continuous ADT was associated with a 2.34-fold increased risk of developing M0 CRPC compared with intermittent ADT, suggesting a potential benefit of intermittent ADT [27]. Furthermore, increased risk of acute myocardial infarction, diabetes mellitus, and bone fractures has been reported with long-term use of ADT [18,19,20]. However, the 5-year risk of cardiovascular events, diabetes, and fractures in intermittent and continuous ADT was compared in 9772 men aged 66 years and older diagnosed with advanced prostate cancer. Results showed that patients who received intermittent ADT had a reduced risk of severe new heart failure (HR 0.62, 95% CI 0.49–0.78, *p* < 0.001) and fracture (HR 0.52, 95% CI 0.38–0.70, *p* < 0.001) [20]. In light of the above, intermittent ADT therapy remains an option to be considered. 

RT and ADT have different adverse event profiles, with RT causing acute adverse events, such as gastrointestinal symptoms like diarrhea and mucous membrane discharge, and urinary symptoms, including urinary frequency and incontinence. Late complications included gastrointestinal bleeding of Grade 3 or more excellent in 4.5%, bladder bleeding in 7.8%, and urinary retention in 7.4%. Late complications of RT are characterized by severe disability [28]. Although ADT also has the aforementioned adverse events, studies have shown that ADT does not increase the risk of cardiovascular events in Japanese and Taiwanese patients [29,30], and the incidence of serious adverse events with ADT may be reduced by selecting the appropriate patient population. Thus, ADT is a potential treatment modality for patients who desire to avoid the adverse events of RT.

Furthermore, basic experiments have shown that the suppression of androgen receptors enhances the migratory potential of prostate cancer cell lines [31,32,33]. In other words, long-term suppression of androgen receptor signaling may induce metastasis, and longer ADT than necessary may lead to poorer oncological outcomes. 

In the Japanese population, intermittent ADT has milder adverse events and a sufficiently better OS than continuous ADT, making it a valuable treatment of postoperative BCR [11]. In addition, it is important to select patients who will benefit from ADT by referring to new biomarkers that predict response to ADT, such as bone turnover markers and prostate cancer positive core rate [34,35].

Noteworthy in this study is that it indicates that strong testosterone suppression may be the only factor that could predict recurrence after salvage ADT. The problem with intermittent therapy is the lack of uniformity in protocols, and establishing the correct criteria for discontinuation of ADT must make intermittent therapy more effective. Previous protocols have adopted the method of stopping ADT when PSA falls below 4 ng/mL [16] or when PSA falls below 4 ng/mL after 8 months of ADT [26]. The adoption of PSA alone as a criterion for discontinuation appears to be a major problem. Therefore, we focused on testosterone levels as a factor for new discontinuation criteria. Numerous studies have shown a correlation between testosterone and prostate cancer outcomes. Pre-treatment serum testosterone levels have been linked to the prognosis of patients who underwent primary ADT [36]. Likewise, in patients with prostate cancer with bone metastases who underwent ADT, the risk of death has also been directly correlated with 6-month serum testosterone levels (HR 1.33, 95% CI 1.05–1.69, *p* < 0.05) [37]. Klotz et al. analyzed 696 patients with prostate cancer undergoing ADT and reported that very low serum testosterone (<0.7 mmol/L) within 1 year after the initiation of ADT was correlated with improved CSS and time to CRPC [38]. The effectiveness of lower testosterone levels during ADT for oncological outcomes has been well documented. Conversely, Yamamoto et al. [39] reported that patients with preoperative testosterone levels below 300 ng/dL had a significantly worse 5-year BCR rate than patients with normal testosterone levels (*p* = 0.035). The significance of testosterone for BCR might change when ADT is used. The results on lower testosterone levels during ADT resulting in better tumor control are consistent with those in previous reports. Trace amounts of testosterone may have contributed to the survival of prostate cancer, and, conceivably, prostate cancer that had not fully regressed may have recurred over time. 

In addition, the criteria for resuming intermittent ADT need to be reconsidered. In previous reports, the protocol was to resume ADT after PSA levels exceeded 10–20 ng/mL [16,26]. One of the analyses of this study revealed that PSA level at the start of treatment is the predictor of CRPC after ADT. It has been reported that ADT for postoperative BCR improved 10-year OS and CSS only when treatment was initiated with PSA < 0.4 ng/mL [40]. Although it was an old case series (1990–1999), it suggests the need for early therapeutic intervention for ADT. In this study, ADT treatment was resumed when PSA exceeded 0.2 ng/mL, which was a much earlier intervention. Early intervention is also indicated in RT. In postoperative RT, early intervention in patients with PSA-DT < 6 months or PSA level < 2 ng/mL improved the OS of patients with BCR after RP [41]. Therefore, whether RT or ADT is chosen, early intervention for postoperative BCR is necessary. This means that earlier restart of ADT may be a factor in better intermittent treatment outcomes.

Several limitations must be acknowledged regarding this study. It was a single-center retrospective study with limited cases. A major limitation is the lack of comparators: ADT has not been directly compared to RT, nor has intermittent ADT been compared to continuous ADT. Although initial treatment was administered at PSA of 0.2 ng/mL, cases of delayed intervention occurred due to patient preference. Moreover, testosterone levels were not always measured at the start of ADT, and the most recent values were used as a reference in such cases. Nevertheless, LH-RH agonists were primarily administered in a 3-month formulation, and considering their half-life, a measurement date error was expected to be acceptable. Another limitation of the intermittent ADT protocol is that few patients received more than a second course, because one course took a long time (2 years) and fewer patients than expected developed PSA recurrence because of their good response to ADT. However, the factor of how many courses of intermittent therapy could be administered is not an important indicator, as this study showed that ADT can be temporarily discontinued for postoperative BCR. Finally, the study did not investigate the impact of ADT on quality of life or the occurrence of adverse events. However, no serious adverse events were observed during the observation period, and most patients completed the treatment, which was expected to be well-tolerated treatment.

## 5. Conclusions

In the Japanese population, ADT is expected to have a certain therapeutic effect on BCR after RARP and is promising for long-term maintenance of CSS and OS. In addition, ADT does not need to be continued throughout life, and intermittent therapy may be an option. One guide to transitioning to intermittent therapy may be to ensure that testosterone declines below the detection limit after the introduction of ADT. This finding needs to be further investigated in future large prospective trials.

## Figures and Tables

**Table 1:** Patients’ characteristics.

Characteristics		All Cases		Intermittent ADT Group *	
No. patients		85		69	
Median age, year (IQR)		69	(65–73)	68	(64–73)
Median follow up, months (IQR)		47	(26–68)	52	(35–73)
Median PSA before RARP, ng/mL (IQR)		7.8	(5.6–12.1)	8.5	(5.6–12.8)
Biopsy Gleason grade group, *n* (%)	1	13	(15)	11	(16)
	2	22	(26)	19	(28)
	3	15	(18)	12	(17)
	4	25	(29)	20	(29)
	5	10	(12)	7	(10)
Clinical T stage, *n* (%)	T2	79	(93)	65	(94)
	T3	6	(7)	4	(6)
Neoadjuvant ADT, *n* (%)	No	66	(78)	53	(77)
	Yes	19	(22)	16	(23)
Surgical margin, *n* (%)	Negative	52	(61)	44	(64)
	Positive	33	(39)	25	(36)
Surgical Gleason grade group, *n* (%)	1	7	(8)	6	(9)
	2	23	(27)	18	(26)
	3	26	(31)	22	(32)
	4	10	(12)	8	(12)
	5	9	(10)	5	(7)
	Not valued	10	(12)	10	(14)
Pathological T stage, *n* (%)	≤2	47	(55)	39	(57)
	≥3	38	(45)	30	(43)
Pathological N stage, *n* (%)	N0	48	(56)	38	(55)
	N1	5	(6)	4	(6)
	Nx	32	(38)	27	(39)
PSA nadir after RARP, ng/mL	<0.008	37	(44)	28	(41)
	≥0.008	48	(56)	41	(59)
PSA at treatment start, ng/mL (IQR)		0.269	(0.241–0.330)	0.265	(0.242–0.323)
Time from RARP to PSA failure, months (IQR)		19	(9–40)	17	(9–33)
Median PSA doubling time, months (IQR)		7	(4–12)	7	(4–11)
Metastasis at treatment start (%), *n*	No	80	(94)	65	(94)
	Yes	3	(4)	2	(3)
	Missing data	2	(2)	2	(3)
Median testosterone level at the end of the first ADT, ng/mL				0.07	(0.03–0.13)
Patients who progressed to CRPC, *n*		5		1	
All-cause death, *n*		4		1	
Prostate cancer-specific death, *n*		2		0	

IQR: Interquartile Range; PSA: prostate-specific antigen; ADT: androgen deprivation therapy; RARP: robot-assisted radical prostatectomy; CRPC: castration-resistant prostate cancer. * Patients who completed initial intermittent ADT.

**Table 2:** Results of univariate Cox proportional hazards regression analysis of CRPC-free survival.

Variables	Univariate Analyses		
	HR	95% CI	*p* Value
Age			
<70 vs. ≥70, years	6.75	0.75–61.14	0.089
PSA			
<10 vs. ≥10, ng/mL	0.02	0.00–48.55	0.333
cT stage			
≤T2 vs. ≥T3	10.28	1.45–73.04	0.020
Biopsy GGG			
≤4 vs. 5	20.98	2.32–189.76	0.007
Neoadjuvant ADT			
No vs. Yes	1.34	0.21–8.65	0.757
pT stage			
≤T2 vs. ≥T3	1.3	0.18–9.23	0.794
Surgical GGG			
≤4 vs. 5	27.49	2.91–260.00	0.004
Surgical margin			
Negative vs. Positive	1.81	0.26–12.92	0.552
PSA nadir			
<0.008 vs. ≥0.008, ng/mL	2.72	0.30–24.35	0.371
PSA at treatment start			
≤0.5 vs. >0.5, ng/mL	9.27	1.53–56.05	0.015
PSA failure			
<10 vs. ≤10, months	5.37	0.57–50.36	0.141
PSA doubling time			
>6 vs. ≤6, months	0.02	0.00–22.76	0.264

HR: hazard ratio; CI: confidence interval; PSA: prostate-specific antigen; GGG: Gleason grade group ADT: androgen deprivation therapy.

**Table 3:** Results of univariate and multivariate Cox proportional hazards regression analyses of biochemical recurrence-free survival.

Variables	Univariate Analysis			Multivariate Analysis		
	HR	95% CI	*p* Value	HR	95% CI	*p* Value
Age						
<70 vs. ≥70, years	1.27	0.53–3.08	0.591			
PSA						
<10 vs. ≥10, ng/mL	1.29	0.54–3.07	0.572			
cT stage						
≤T2 vs. ≥T3	0.49	0.07–3.68	0.489			
Biopsy GGG						
≤4 vs. 5	0.75	0.17–3.24	0.700	1.21	0.15–9.85	0.857
Neoadjuvant ADT						
No vs. Yes	1.49	0.60–3.72	0.393			
pT stage						
≤T2 vs. ≥T3	0.87	0.37–2.09	0.763			
Surgical GGG						
≤4 vs. 5	2.26	0.51–10.07	0.287			
Surgical margin						
Negative vs. Positive	1.66	0.70–3.96	0.251			
PSA nadir						
<0.008 vs. ≥0.008, ng/mL	0.75	0.31–1.78	0.513			
PSA at treatment start						
≤0.5 vs. >0.5, ng/mL	1.59	0.47–5.40	0.461			
PSA failure						
>10 vs. ≤10, months	1.01	0.42–2.46	0.977			
PSA doubling time						
>6 vs. ≤6, months	0.80	0.33–1.93	0.617	0.82	0.27–2.52	0.731
Testosterone						
≤0.03 vs. >0.03, ng/mL	4.76	1.06–21.37	0.041	5.10	1.10–23.59	0.037

HR: hazard ratio; CI: confidence interval; PSA: prostate-specific antigen; GGG: Gleason grade group; ADT: androgen deprivation therapy.

## Data Availability

The data presented in this study are available within the article.
